# First report on the presence of huanglongbing vectors (*Diaphorina citri* and *Trioza erytreae*) in Ghana

**DOI:** 10.1038/s41598-023-37625-9

**Published:** 2023-07-13

**Authors:** Owusu F. Aidoo, Fred K. Ablormeti, Kodwo D. Ninsin, Akua K. Antwi-Agyakwa, Jonathan Osei-Owusu, William K. Heve, Aboagye K. Dofuor, Yovanna L. Soto, George Edusei, Angelina F. Osabutey, Frederick L. Sossah, Clement O. Aryee, Olufemi J. Alabi, Mamoudou Sétamou

**Affiliations:** 1 Department of Biological Sciences, University of Environment and Sustainable Development, PMB, Somanya, E/R Ghana; 2 Council for Scientific Industrial Research, Oil Palm Research Institute, Coconut Research Programme, P. O. Box 245, Sekondi, Ghana; 3 Cocoa Research Institute of Ghana, New Tafo, E/R Ghana; 4 Department of Physical and Mathematical Sciences, University of Environment and Sustainable Development, Somanya, Ghana; 5 Texas A&M University-Kingsville Citrus Center, Weslaco, 78599 USA; 6 Presbyterian University, Ghana, Abetifi-Kwahu, Eastern Region Ghana; 7 Department of Plant Pathology and Microbiology, Texas A&M AgriLife Research and Extension Center, Weslaco, TX 78596 USA

**Keywords:** Biological techniques, Ecology, Evolution, Genetics, Molecular biology, Zoology

## Abstract

As significant threats to global citrus production, *Diaphorina citri* (Kuwayama; Hemiptera: Psyllidae) and *Trioza erytreae* (Del Guercio; Hemiptera: Triozidae) have caused considerable losses to citrus trees globally. *Diaphorina citri* vectors “*Candidatus* Liberibacter asiaticus” and “*Ca.* L. americanus”, whereas *T. erytreae* transmits “*Ca.* L. africanus” and “*Ca*. L. asiaticus”, the pathogens responsible for citrus greening disease or Huanglongbing (HLB). Though HLB is a destructive disease of citrus wherever it occurs, information on the occurrence and geographical distribution of its vectors in Africa is limited. In recent surveys to determine if HLB vectors are present in Ghana, we observed eggs, nymphs, and adults of insects suspected to be *D. citri* and *T. erytreae*. Using morphological traits and DNA analyses, the identity of the suspected insects was confirmed to be *D. citri* and *T. erytreae*. Individuals of *D. citri* and *T. erytreae* were examined using qPCR for *C*Laf, *C*Lam, and *C*Las, but none of them tested positive for any of the Liberibacter species. Herein we report, for the first time, the presence of *D. citri* and *T. erytreae* in Ghana (West Africa). We discuss the implications of this new threat to the citrus industry to formulate appropriate management strategies.

## Introduction

The Asian citrus psyllid *Diaphorina citri* Kuwayama, 1908 (Hemiptera: Psyllidae) and African citrus triozid *Trioza erytreae* (Del Guercio, 1918) (Hemiptera: Triozidae) are the most devastating pests of citrus. Their potential for significant negative impacts on citrus production and productivity is high due to international trade and the movement of host plant materials across borders^1,2^. *Diaphorina citri* and *T. erytreae* are native to southern Asia and Africa, respectively^3,4^. However, as of November 2022, each of them has been reported from at least 25 countries worldwide^5–7^. In Africa, *D. citri* occurs in Kenya and Zanzibar^8^, and more recently, in Nigeria, Ethiopia, and Benin^9–11^. Outside Africa, *T. erytreae* has been reported from Saudi Arabia, Yemen, Spain and Portugal^12^. Maximum Entropy and Climate Change Experiment modeling of the potential global distribution of *D. citri* and *T. erytreae* in the world suggest that parts of uninvaded regions of Africa have climate suitable areas for the proliferation of both *D. citri* and *T. erytreae*^5,7^.

*Diaphorina citri* and *T. erytreae* feed on citrus and non-citrus species. Specifically, a wide variety of plants, especially those in the family Rutaceae, serve as hosts for *T. erytreae* and *D. citri.* The pests are highly polyphagous, with each having at least 23 non-citrus host plants^13,14^. The pests attack many economically important citrus species, including grapefruit, lemon, lime, and sweet oranges. However, the most preferred host plants for *D. citri* are the orange jasmine *Murraya paniculata* (L.) Jack., and the curry leaf plant *Bergera koenigii* (L.)^15^. Orange jasmine is a commonly grown ornamental plant in residential areas for landscaping, and has therapeutic properties^16^. In contrast, *T. erytreae* prefers *Citrus limon* (L.) to other host plants^17,18^.

The development of the vectors goes through five nymphal instars. The adults of *D. citri* have speckled brown wings and measure between 2.7 and 3.3 mm in length^19^. The females of *D. citri* may lay a mean of 40 eggs per flush shoot per day^20^. The freshly laid eggs of *D. citri* are pale but change into yellow and orange with two unique red eye spots at maturity^19^. *Diaphorina citri* lays on tips of developing shoots on and between unfurling leaves^21^. *Trioza erytreae* is about 2.40 mm long, with females being slightly larger than males^18^. *Trioza erytreae* adults start pale but gradually darken afterwards to a light brown. *Trioza erytreae* female lays eggs on the margins and along the midribs of young and tender leaves^22^. The average number of eggs laid by *T. erytreae* females per day ranges from 17 in the cold to 39 in the warm seasons, respectively^23^. When feeding on leaves, adults rest at a body angle of about 35° and 45° for *T. erytreae* and *D. citri*, respectively^12,19^.

Both *D. citri* and *T. erytreae* cause direct and indirect damage to citrus. Feeding activities of *D. citri* nymphs and adults lead to substantial uptake of plant sap and may induce sooty mold development due to honeydew production on infested flush shoots. Sooty mold affects the photosynthetic activities of the citrus trees, thereby reducing their productivity^24^. Direct foliage feeding by *T. erytreae* results in stunted and seriously deformed leaves showing signs of pit-like galls^23^. Although direct feeding damage of psyllids may result in a loss of plant vigor, indirect damage via vectoring of HLB pathogens is the most economically important. All over the world, HLB is caused by the phloem-limited bacteria “*Ca.* L. asiaticus” (*C*Las) and *Ca.* Liberibacter americanus (*C*Lam) transmitted by *D. citri*^25,26^. In contrast, in Africa, especially East and South Africa, *T. erytreae* represents an economically significant threat to the citrus industry because of its ability to vector "*Ca.* Liberibacter africanus" (*C*Laf), the causal agent of the African HLB. However, a recent study by Ajene et al.^27^ showed field populations of *T. erytreae* carrying *C*Las, and Reunaud et al.^28^ demonstrated that it also can efficiently transmit *C*Las. Unlike the HLB caused by *C*Laf, the HLB caused by *C*Las is the most destructive pathogen of all citrus in the world^28^.

*D. citri* have the ability to fly both long and short distances. However, its propensity to engage in long-distance flights, as well as the length and distance flown, are all affected by high heat conditions, regardless of the humidity level^29^. Nevertheless, the most extended flight distance and duration are shorter in females than males^30^. Additionally, *D. citri* routinely migrates between managed and unmanaged groves, covering a distance of about 60–100 m toward managed groves^31^. Because *D. citri* eggs are laid solely on young flush and nymphs develop primarily on young plant parts, population variations of this pest are highly correlated with the growth of new and young flush of the host plant^32,33^. According to Catling^23^, *T. erytreae* has weak dispersal powers and are incapable of sustaining flight for long hours. In contrast, Samways and Manicom^34^, showed that *T. erytreae* had a good dispersal ability and could invade orchards to locate new flush shoots.

Citrus is one of the most widely grown fruits worldwide due to its high demand and positive impact on food and nutritional security^35^. In terms of global trade value, it is one of the most valuable fruits^36^. The commonly known species of commercial importance are sweet oranges (*Citrus sinensis*), lemons (*C. limon*), limes (*C. aurantifolia*), grapefruits (*C. paradisi*), and tangerines (*C.* *reticulata*). Sweet oranges are the major species grown globally and represent more than half of the global citrus output. Annual global production of citrus was estimated at 158 million tonnes in 2020, with sweet oranges contributing to more than half of the world's total^37^. Africa produced 9,756,176 metric tons of sweet oranges from an estimated 514,573 ha in 2020, while production in Ghana was at 697,637 metric tons from an estimated 17,983 ha^37^. The health benefits of sweet oranges and other citrus fruits are well documented, especially in providing vitamin C, carotenoids, and polyphenols. Most citrus fruits in many countries, including Ghana, are consumed locally as fresh products^38^.

Early detection of *D. citri* and *T. erytreae* would help develop strategies and manage the pests and diseases they transmit, slowing down their spread and preventing them from becoming established. Despite reports of the presence of the invasive and deadly *D. citri* in neighboring Nigeria and Benin^10,11^, there have been no previous investigations to determine the existence of *D. citri* in Ghana. Given the economic importance of citrus production in Ghana, we conducted surveys for citrus commodity pests in Ghana to determine the presence of *D. citri* and *T. erytreae* and, if found, to test the samples for Liberibacter species.

## Materials and methods

### Sample collection

To establish the presence of *D. citri* and *T. erytreae* in Ghana, surveys of residential areas and citrus orchards in the Volta Region, Ghana (Table 1), were conducted from April to November 2022. The main roads in each town were chosen for sampling. All encountered *Citrus* and *Murraya* species were visually inspected for the presence of *D. citri* and *T. erytreae*.

**Table 1:** Field characteristics of Asian citrus psyllid (*D. citri* Kuwayama) and African citrus triozid (*T. erytreae* Del Guercio) individuals suspected of being present in samples taken from different sites in Volta Region, Ghana.

Species	Locality	Host plant	Purpose	Latitude	Longitude	Elevation	Adults	Nymphs	Eggs	Galls
*Diaphorina citri*	Ahoe	*Murraya paniculata*	Ornamental	6.60147	0.46882	173	33	0	0	–
	Dzavieme	Citrus	Backyard garden	6.60316	0.46894	174	0	0	0	–
	Hevi	*Murraya paniculata*	Ornamental	6.60176	0.47119	172	36	86	101	–
	Power house	*Murraya paniculata*	Ornamental	6.58339	0.48081	112	11	55	0	–
	Mawuli Estate	Citrus	Backyard garden	6.59262	0.4725	142	0	0	0	–
	Dome	*Murraya paniculata*	Ornamental	6.60838	0.47892	147	23	33	55	–
*Trioza erytreae*										
	Awate-Todzi	*Triclisia subcordata* (Oliv.)	Backyard garden	6.86823	0.24543	227	152	233	355	Yes

Each backyard hedge (Fig. S1) with a host plant was visually examined for *D. citri* adults and symptomatic leaves (Fig. S2). Expanding flush shoots and at high densities on the stems are where *D. citri* nymphs are attached (Fig. S3). In contrast, *T. erytreae* nymphs are usually found on the underside of the leaves (Fig. S4). To collect the nymphal stages from the plants, we thoroughly searched for the immature stages on the stems and new tender shoots. Inspection of host plant was further aided by feeding damage, such as 'epinasty', and distortion of young and fragile leaves due to the sap-feeding of *D. citri* nymphs and adults, and the presence of the white waxy excretions from the nymphs (*D. citri*)^10,19^. In addition, *T. erytreae* pit-like galls were used to detect the presence of the triozid^18^. In case adult insects were found, they were aspirated into plastic vials containing 95% ethanol, and nymphs were brushed into similar vials using a fine camel brush. The host plant observed was recorded for each location. Moreover, the coordinates of each sample location were recorded using a handheld Garmin eTrex^®^ 32x device.

### Identification of the suspected *D. citri* and *T. erytreae*

#### Morphological identification

The insects were first identified visually using morphological characteristics^4^, and then a subset of samples was sent to the Texas A&M University Kingsville Citrus Center (TAMUK-CC) in Weslaco, Texas, USA, for additional morphological and molecular analyses. The suspected insects underwent a thorough morphological characterization at TAMUK-Entomology CC's laboratory, where they were compared to preserved reference voucher specimens. We used the morphological characteristics reported by Mead^4^, Yang^39^, and OEPP/EPPO^40^ to positively identify *D. citri* nymphs and adults. Moreover, Aidoo et al.^18^ and Cocuzza et al.^12^, reports on *T. erytreae* morphometry were used for their identification. The voucher specimens were kept at TAMUK-CC and the Central Laboratory of the University of Environment and Sustainable Development, Somanya, Ghana.

#### Nucleic acid isolation and PCR

In this study, we used the method described by Dellaporta et al.^41^ to extract total nucleic acids (TNA) from both adults and nymphs of the psyllids. NanoDrop 2000 series spectrophotometer (Thermo Fisher Scientific Inc., Waltham, MA, USA) was used to measure the concentration and purity of the TNA extracts before they were frozen at 20 °C for later use. Two microliters (uL) of each sample was utilised as template in a 25 μL polymerase chain reaction (PCR) using the PrimeSTAR GXLDNA Polymerase, its recommended reagents, and the Rapid Protocol (Takara Bio USA, Inc., Mountain View, CA). We targeted 821-bp and 708-bp segments of the *mtCOI* coding region in the *D. citri*^42^ and *T. erytreae*^43^ using the primer pair DCITRI COI-L and DCITRI COI-R and Te-6U30 and Te-720L26, respectively. Positive controls consisted of DNA samples taken from TAMUK-lab-reared CC's ACPs. The 100–2000 bp Wide-Range DNA Ladder (Takara Bio USA, Inc.) and the amplified products were run on ethidium bromide-stained 1% agarose gels and then visualised using a UV-transilluminator.

#### Cloning and sequencing

Zymoclean Gel DNA Recovery Kit was used to cut out and gel-elute the appropriate size DNA bands from the sample and the target (Zymo Research, Irvine, CA). After purification, the recovered DNA was cloned into the pJET1.2/blunt vector one at a time using the CloningJET PCR Kit (Thermo Fisher Scientific). Transforming chemically competent DH5 *Escherichia* *coli* cells with the ligation products yielded two to three plasmids per cloned DNA amplicon that were PCR-verified to be the correct size (Sigma-Aldrich, St. Louis, MO). By using the Sanger sequencing technique and primers designated as pJET1.2 F and pJET1.2 R, each plasmid sample was sequenced in both directions (ELIM BIOPHARM, Hayward, CA, USA).

#### Bioinformatic analysis

The pJET1.2 vector sequences were removed using VecScreen (https://www.ncbi.nlm.nih.gov/tools/vecscreen/). Each sample's forward and reverse sequences were entered into the CAP contig assembly function of the BioEdit software^44^ to generate a consensus sequence. To determine which species each consensus sequence belonged to, BLASTn analysis^45^ was performed on all the sequences of the psyllids. Multiple sequence alignments were generated between the sequences derived in this study and those obtained from GenBank using the MUltiple Sequence Comparison by Log-Expectation alignment program (http://www.ebi.ac.uk/Tools/msa/muscle/). The sequences were chosen because they are representative of the diversity of the taxonomic groups studied. The sequence identity matrices and phylogenetic analyses were calculated using the maximum likelihood approach in MEGA version 7.0^46^, which was applied to the gene-specific alignment data. Instead of using tables of pairwise sequence identity scores, which are commonly used for this purpose, it is recommended to use the Sequence Demarcation Tool (SDT), which displays pairwise identity scores using a color-coded matrix^47^. This makes it easier to gain insights into the overall relationships between sequences in a dataset. Moreover, we calculated the pairwise using SDT in this study. *Trioza* species considered in this study included those from Percy et al.^48^ and Khamis et al.^49^.

#### Tests for *Ca*. Liberibacter spp.

TaqMan Multiplex Real-Time PCR tests were done on an ABI 7500 Fast Thermocycler (Thermo Fisher Scientific Inc., Waltham, MA) or a SmartCycler II (Cepheid, Sunnyvale, CA) and the DNA extracts were assayed for *C*Laf, *C*Lam, and *C*Las as described^50–52^. All reactions included a standard set of positive and negative DNA controls, as well as a non-template water control. At a cycle threshold (Ct) of ≤ 37, it was determined that a sample was positive for the presence of a given bacterium.

### Ethical standards

This article does not contain any studies with human participants or animals performed by any of the authors.

## Results

### Field detection and morphological identification

**Figure 1:**
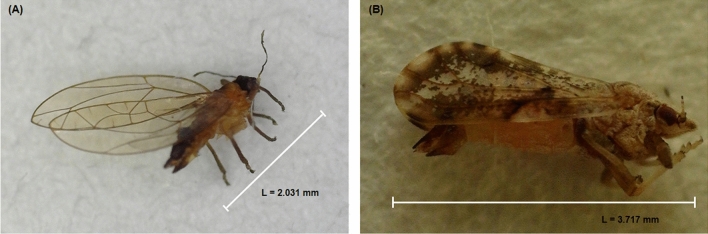
Female adults of the African citrus triozid (*Trioza erytreae* Del Guercio) (**A**) and the Asian citrus psyllid (*Diaphorina citri* Kuwayama) (**B**) detected in different locations (Table 1) in Volta Region, Ghana.

The altitudes of all locations investigated ranged from 112 to 174 m above sea level (m.a.s.l). The field-collected insects were morphologically identified as *D. citri* and *T. erytreae* using previously published features^4,12,18,39,40^, and by comparison with voucher specimens (Fig. 1).

Both the TAMUK-CC Entomology Laboratory in Weslaco, Texas, and the University of Environment and Sustainable Development in Somanya, Ghana, now hold voucher specimens of these samples. Four of seven locations had adults *D. citri* feeding on mature and/or young growing oranges jasmine leaves during the study. We found that *D. citri* was more established and reasonably widespread in Volta Region than *T. erytreae*, given the vast dispersion of the positive detection in different locations and the observation of developing nymphs and eggs at three different sites (Table 1).

### Molecular detection

A subset of six adults each of *D. citri* and *T. erytreae* were chosen at random to represent the geographical diversity of the sampled insects, and their gene-specific DNA amplicons were found to be of the predicted sizes (Table 2).

**Table 2:** Primers used for the amplification of gene segments of the Asian citrus psyllid (*D. citri*) and African citrus triozid (*T. erytreae*).

Primer name	Sequence (5′–3′)	(bp)	Host/gene target	Source
DCITIR COI-L	AGGAGGTGGAGACCCAATCT	834	*D. citri*/*mtCOI*	Boykin et al.^42^
DCITRI COI-R	TCAATTGGGGGAGAGTTTTG			
Te-6U30	ATTTTTAAGCACTAATCATAAAATTATTGG	708	*T. erytreae* /*mtCOI*	Pérez-Rodríguez et al.^43^
Te-720L26	TATACTTCAGGATGTCCAAAAAATCA			

There was a total of six sequences for *D. citri* COI; four using DCITRI COI-L/DCITRI COI-R and two using Te-6U30/Te-720L26. The *T. erytreae* samples had six sequences. *D. citri* sequences obtained with primers DCITRI COI-L/DCITRI COI-R showed significant matches (100.0% nt identical; 100% query coverage; E-value 0.0) using BLASTn between these sequences and corresponding gene-specific sequences of *D. citri* that are deposited in GenBank from different countries. When *T. erytreae* primers Te-6U30/Te-720L26 were used to amplify the COI gene of *D. citri*, the results also revealed a significant (99.30–99.58% nt identical; 100% query coverage; E-value 0.0) matches with sequences of *D. citri* from other countries. *Trioza erytreae* sequences obtained with primers Te-6U30/Te-720L26 showed a significant (100% nt identical; 100% query coverage; E-value 0.0) matches using BLASTn between these sequences and corresponding gene-specific sequences of *T. erytreae* that are deposited in GenBank from different countries.

### Phylogenetic analysis

**Figure 2:**
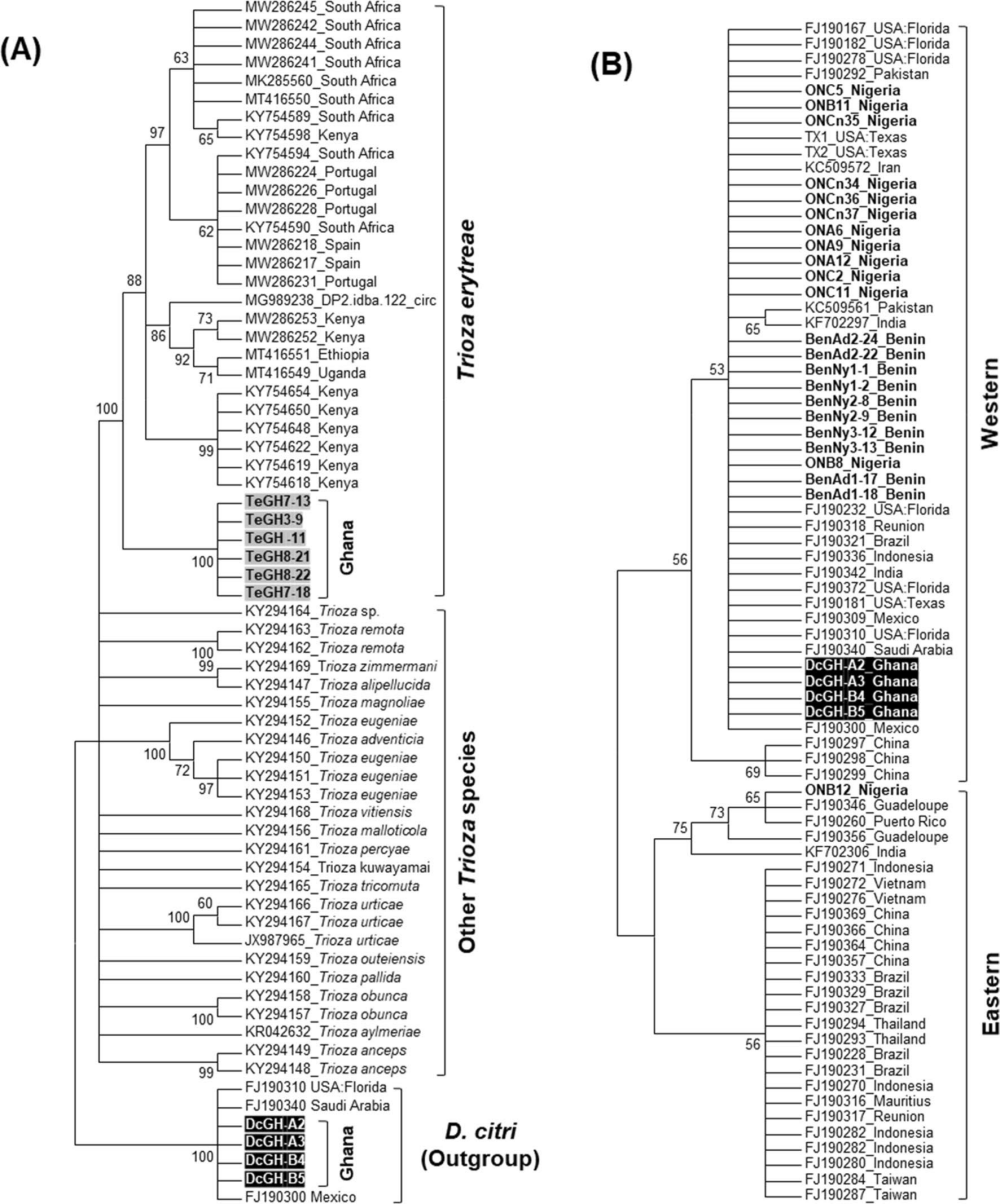
(**A**) Maximum Likelihood (ML) phylogenetic trees depicting the evolutionary relationships between adult individuals of *Trioza erytreae* and other *Trioza* species based on partial sequences of the maternally inherited *mtCOI* gene; OR036870–OR036875 derived in this study; others from GenBank. The corresponding sequences of *Diaphorina citri* were included as outgroup; (**B**) Phylogenetic placement of the *D. citri* isolates from Ghana into the previously described ‘Western clade’ of the insect species; OR036866–OR036869 obtained in this study; others from GenBank. The General Time Reversible and the Tamura 3-parameter models were determined as models with the lowest BIC (Bayesian Information Criterion) scores, respectively, for the figure (**A**) and (**B**) datasets and they were therefore used in the ML phylogenetic analysis for each of the gene-specific sequences (with 1000 bootstrap replications). Branches with < 50% bootstrap support were collapsed for each tree. The ML trees were generated using MEGA 7.0 (Kumar et al.^46^, https://www.megasoftware.net).

Maximum likelihood (ML) phylogenetic analysis of each gene-specific sequence was performed, and the Tamura 3-parameter model was shown to have the lowest Bayesian Information Criterion (BIC) scores. As expected, it was predicted that the Ghana *mtCOI* sequences of *T. erytreae* (OR036870- OR036875) and *D. citri* (OR036866- OR036869) fall inside the *T. erytreae* and *D. citri* clade of the psyllid ML trees (Fig. 2). Our analysis revealed that *T. erytreae* samples from Ghana clustered with samples from other countries. After further analysis, it was shown that the *mtCOI* sequences unique to *D. citri* clustered strongly into the Western clade, which consists of populations from many countries. In addition, the *D. citri* samples from Ghana may likely represent a separate introduction event into Africa considering how distant they are from the other samples of Benin and Nigeria.

### Color-coded pairwise identity

**Figure 3:**
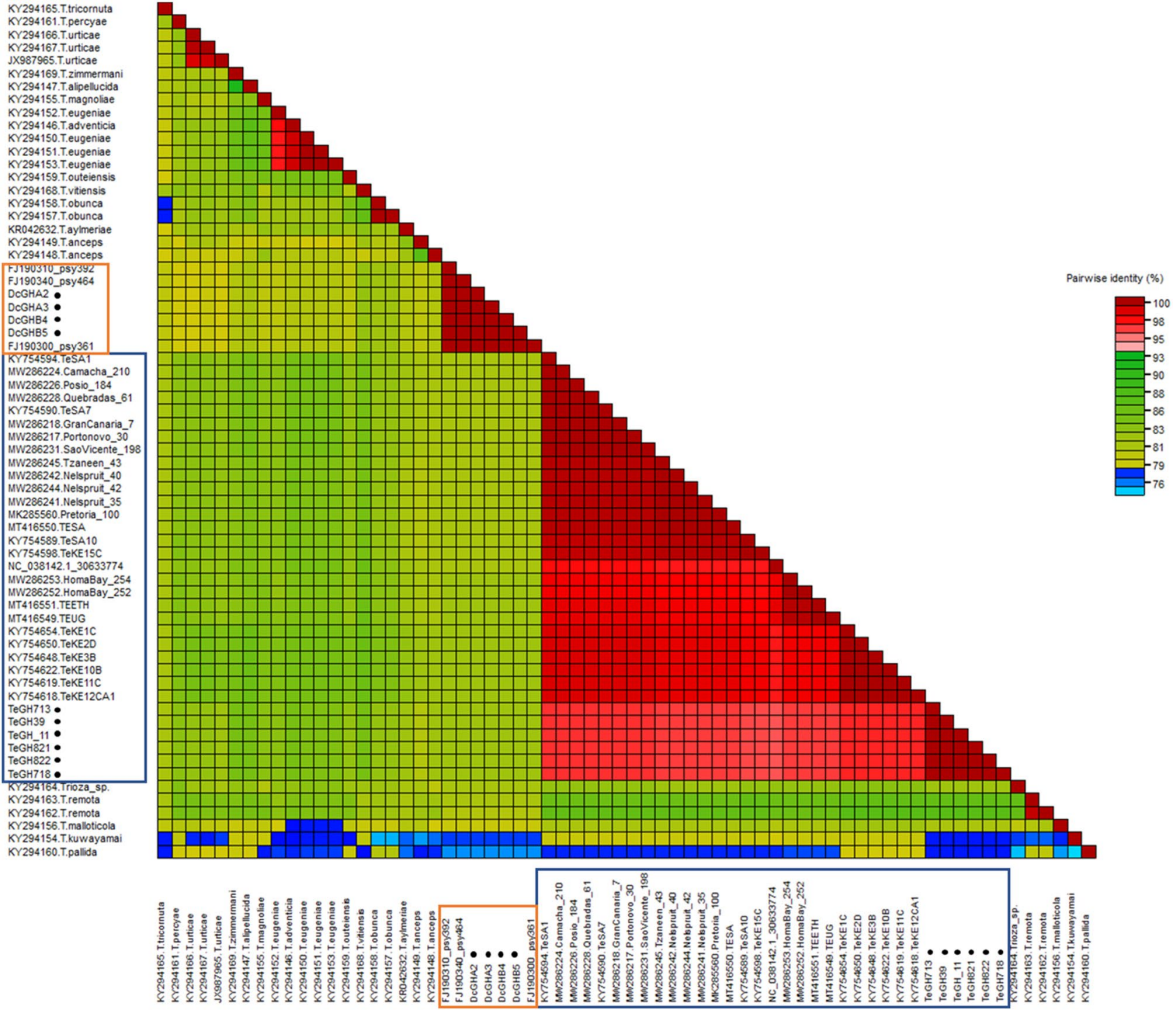
Color-coded pairwise identity matrix generated from partial sequences of the maternally inherited *mtCOI* gene of isolates of *Trioza erytreae* (blue box), other *Trioza* species and *Diaphorina citri* (orange box). The sequences obtained in this study from Ghana are denoted with black dots. The matrices were generated using the Sequence Demarcation Tool (SDT) v.1.2 (Muhire et al.^47^; http://web.cbio.uct.ac.za/).

Possible demarcation criteria for the *T. erytreae* is assigned and compared to other species of *Trioza*. Based on the analyzed partial *mtCOI* sequences, the *T. erytreae* samples from Ghana appear to be distant relatives of the species *T. erytreae* based on the combination of sequence variation and geographical segregation (Fig. 3). *Trioza erytreae* collected from Ghana belong to the same demarcation as *T. erytreae* from other countries when analyzed using the SDT.

## Discussion

For the first time, we report the presence of *D. citri* and *T. erytreae* in Ghana using both morphological and molecular techniques. However, there have been recent reports of *D. citri* in Nigeria (West Africa)^10^ and other African countries, such as Ethiopia^9^, Tanzania^53^, Kenya^8^ and Benin^11^. In response, many citrus-producing areas in eastern and southern African countries have increased the intensity of their pest surveillance and monitoring^10^. Herein, we initiated this study to ascertain the status of *D. citri* in Ghana to make early detection (if present) to inform concerted management efforts in Ghana and across sub-Saharan Africa. In addition, preventing an invasion is almost always cheaper than managing an invasive species once it has already entered an area^54^. Moreover, this was done due to the dangers of transporting plants around the world and the fact that citrus has been grown in Ghana for centuries.

*Diaphorina citri* in Ghana was confirmed at elevations of less than 200 m.a.s.l. According to Holford et al.^55^, in Indonesia and Bhutan, high altitudes above 1000 m.a.s.l limit the incidence and occurrence of HLB and *D. citri*, respectively. The entire country (i.e., Ghana) has an elevation of less than 1000 m.a.s.l. The mean annual temperatures of Ghana range between 24 to 30 °C, though it can be as low as 18 °C in the south and as high as 40 °C in the north^56^. *Diaphorina citri* prefers warm and dry climates and suitable temperatures for development range between 25 and 28 °C^57^. It can also tolerate temperatures above 40 °C^29^. However, high temperatures may decrease the flight capacity of the pest^29^. Average annual rainfall in the north of Ghana is below 1000 mm, whereas it averages approximately 2000 mm in the south^56^. Given the suitable environmental conditions in Ghana for *D. citri*, it is possible that the pest is widely distributed or can spread to other citrus-growing regions in the country.

*Trioza erytreae* was identified in one location in the Volta Region during the survey. A temperature-based phenology model study on *T. erytreae* predicted that optimum temperatures for the pest ranged from 20 to 25 °C^58^. The temperature in Ghana suggests that *T. erytreae* has the potential to spread in the country. However, it will be easier to manage *T. erytreae* than *D. citri* in Ghana because of its environmental requirements. In the future, it will be imprudent to ease off in developing management techniques aimed at preventing and managing agricultural pests because of their potential to adapt to the changing climates in many regions^59^.

A recent study, which used a species distribution model of suitability, concluded that most tropical Africa has a suitable climate for spreading *D. citri* and *T. erytreae*^5–7^. As a result of their research, they created a predictive niche map showing that many West, East, and Central African countries, including Ghana, are at high risk for *D. citri* establishment. Considering this, the presence of *D. citri* in Ghana demonstrates the severe threat posed by this invasive species to Ghana and other African countries where the pest is absent. The *mtCOI* of *D. citri* has been widely used in genetic variation and population structure studies^60–62^ because of its adaptability in diversifying insect populations across different geographical areas.

The presence of *D. citri* is of particular concern in Ghana, where agriculture is the backbone of the economy. Moreover, *D. citri* has risen to become a global threat to the viability of citrus businesses wherever the pest and the disease it transmits occur^25^. Agriculture remains a critical tool for sustainable development in many countries across sub-Saharan Africa, providing hundreds of millions of rural poor with new avenues out of poverty through smallholder farming, work in high-value crop production, entrepreneurial endeavors, and employment in rural and non-farming sectors. Despite efforts to ensure sustainable crop production, pests, and diseases persistently pose a threat on the continent. One such pest is *T*. *erytreae*, which is limited to Africa, the Middle East, and Europe^5,6^. The presence of *D. citri* in Ghana, which has a wide distribution in the North, Central, and South America, demonstrates that the combined effects of the HLB vectors could worsen the present losses associated with citrus pests. *Trioza erytreae* is heat-sensitive and develops best in the cooler highlands, while the Asiatic strain is believed to be more virulent and damaging overall^63^. This, however, suggests that the presence of HLB could threaten the sustainable production of citrus in Ghana due to the suitable climate suitable areas in the country^7^.

Huanglongbing causal agents can be disseminated via grafting and vegetative propagation. However, *D. citri* is implicated in much of their long-distance and within orchard distribution. *Diaphorina citri* can be transported over long distances by moving citrus materials like seedlings and alternate host plants. However, the psyllid may also move over long distances, and the prevailing wind direction and intensity can facilitate distance movement^64–66^. *Diaphorina citri* is primarily responsible for the introduction and subsequent spread of the Asian type of HLB in various parts of the world, including Brazil^67^, Texas^68^, China^69^, and California^70^.

In this study, *D. citri* was obtained from an alternate host plant (*M. paniculata*). Alternate hosts play a critical role in the dispersal and management of invasive pests^71,72^. *Murraya paniculata* is grown as an ornamental tree or hedge due to its durability, adaptability to a wide variety of soil, and suitability for larger hedges. In addition, the plant has antimicrobial, antioxidant, red blood cell membrane stabilization, and anti-inflammatory properties and is used to treat many diseases^73^. As a result, it has been used in many parts of the world for symptoms such as nausea, vomiting, constipation, diarrhea, stomach discomfort, headache, fluid retention, and clot formation^16^. In Ghana, the plant is mainly grown for its therapeutic uses, beautification and as a hedge.

### Implication for management

Although the psyllids were collected from non-citrus host plants in Volta Region, Ghana, nationwide surveys targeting citrus and non-citrus host plants are urgently needed to define the extent of the spread in Ghana. Effective management of psyllids and HLB can only be achieved through a thorough assessment of the distribution of the pest and the disease it transmits. In addition, identifying localities that are free of infestation and where clean nursery programs can be established would offer some level of management against the psyllids. The removal of alternate host plants can be facilitated by a better understanding of areas where these plants are planted, which may also reveal the presence of previously unknown reservoirs of *Ca.* Liberibacter species. The farming practices in Ghana predominantly revolve around subsistence farming, which contradicts the effective implementation of recommended practices for managing HLB. These practices include establishing clean nurseries, implementing intensive psyllid management across larger contiguous blocks, and more. To effectively manage the psyllids, there is a need for regular inspection of citrus and non-citrus host plants at the Ghanaian ports of entry, and intercepted psyllids should be tested for HLB^5,74^. Given the confirmation of both *D*. *citri* and *T*. *erytreae*, it is essential to evaluate the existing distribution of *D*. *citri* not only in Ghana but also in neighboring regions. Coordinated regional management initiatives should be initiated promptly to eliminate these destructive pests before they become endemic or spread *C*Las, *C*Lam, and *C*Laf, which could have severe consequences throughout the region. If found in citrus, efforts will be required to identify optimal crop management, such as crop rotation, intercropping and planting time. Also, developing resistance varieties, such as genetically engineered insecticidal types, could help control the psyllid populations in an environmentally friendly manner because resistance in field populations of *D. citri* has been reported in citrus groves^75,76^. In this study, it is important to note that *D*. *citri* was also sequenced using the *T*. *erytreae*-specific primer. This *T*. *erytreae*-specific primer used for *D*. *citri* sequencing has GenBank accession numbers OR036900 and OR036901. This information is valuable for identifying psyllids at entry points, thereby enhancing detection capabilities. Furthermore, Wenninger et al.^77^ reported that *D*. *citri* adults can exhibit various colorations, including gray/brown, blue/green, and orange/yellow forms, with noticeable differences in abdominal coloration. To facilitate early detection of *D*. *citri*, a combination of morphological identification and subsequent molecular studies can be useful in identifying psyllids present on planting materials at entry points.

## Supplementary Information

Supplementary Figures.

## Data Availability

The sequences obtained in this study have been deposited in NCBI GenBank with the following accession numbers: OR036866–OR036875 and OR036900–OR036901.
