# Supplementary material for: First report on the presence of huanglongbing vectors (Diaphorina citri and Trioza erytreae) in Ghana
Source: Sci Rep. 2023 Jul 13;13:11366. doi: 10.1038/s41598-023-37625-9 (PMC10344884; doi:10.1038/s41598-023-37625-9)
Supplement: Supplementary file 1 — Supplementary Figures. [file 41598_2023_37625_MOESM1_ESM.docx]

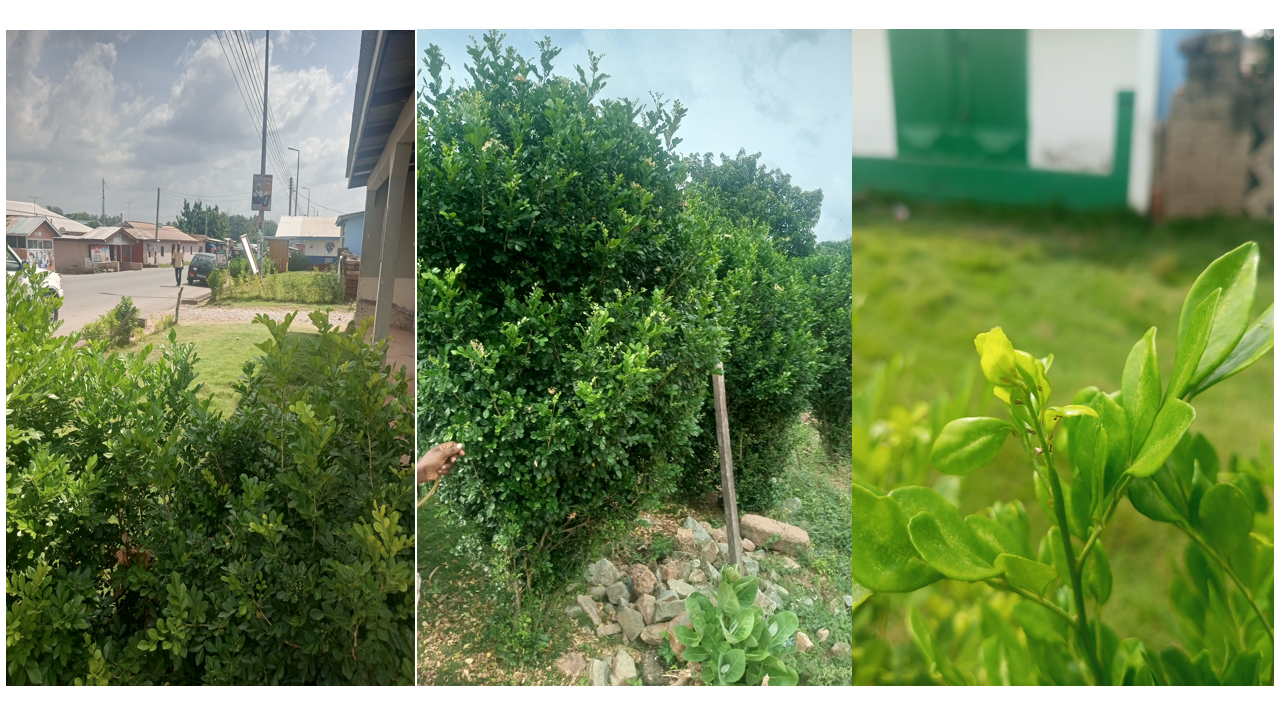
Fig. S1. *Murraya paniculata* Linnaeus being used as a hedge in the Volta Region, Ghana.


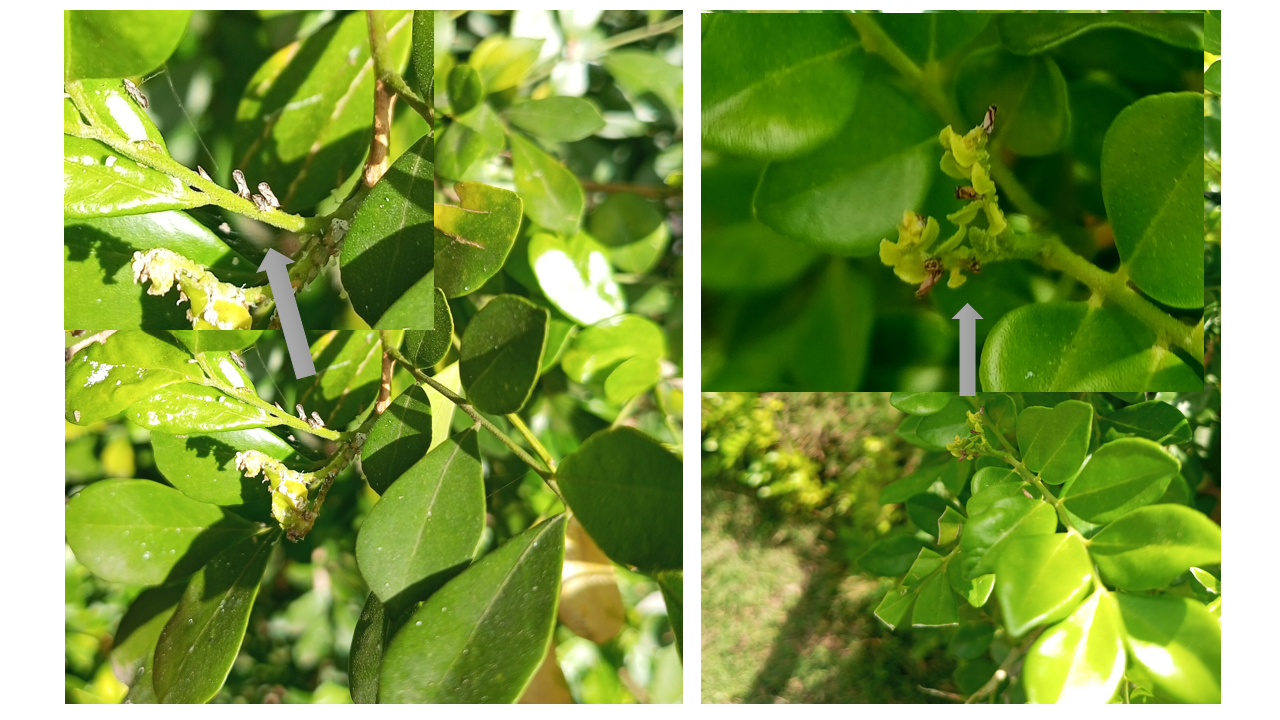
 Fig. S2. Adult of the suspected Asian citrus psyllid (*Diaphorina citri* Kuwayama) observed in Orange Jasmine (*Murraya paniculata* Linnaeus) in one of the locations in Ho, Volta Region, Ghana.


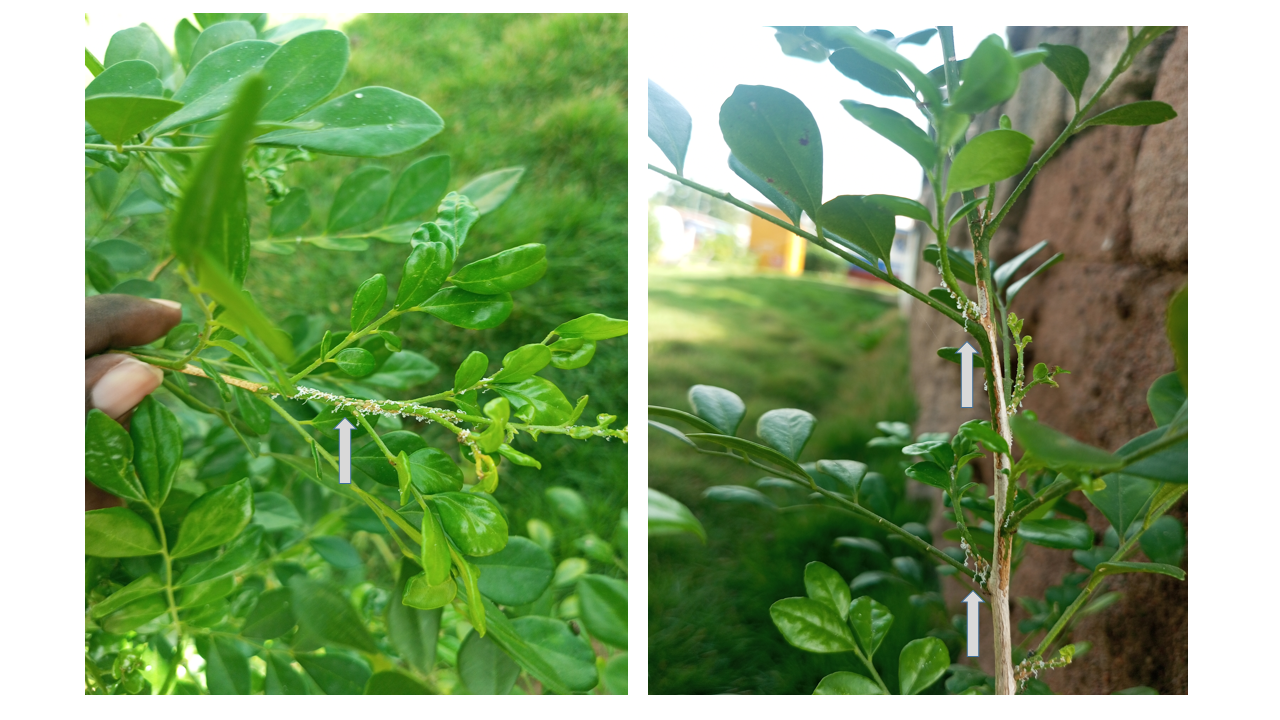
Fig. S3. Nymphs of the suspected Asian citrus psyllid (*Diaphorina citri* Kuwayama) observed in Orange Jasmine (*Murraya paniculata* Linnaeus) in one of the locations in Ho, Volta Region, Ghana.


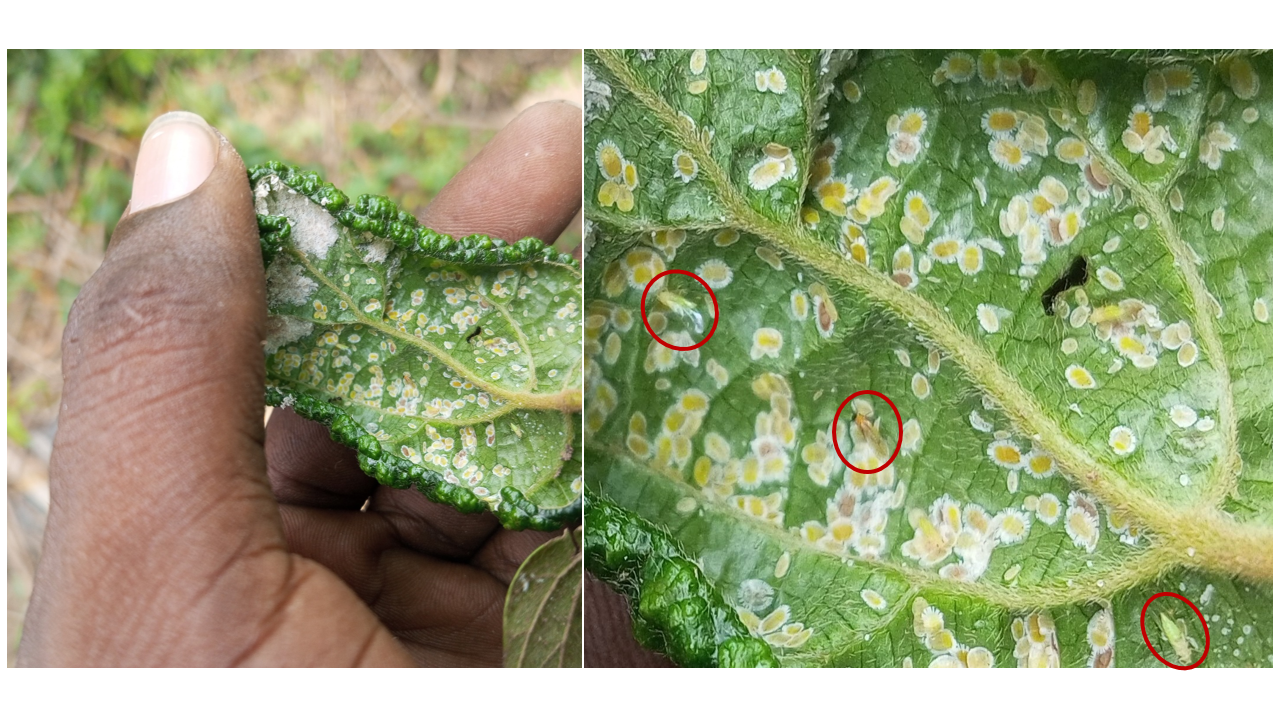
Fig. S4. Adults (circled) and nymphs suspected to be African citrus triozid (*Trioza erytreae* Del Guercio) observed in *Triclisia subcordata* (Oliv.) in one of the locations in Volta Region, Ghana.
